# Disaster Reintegration Model: A Qualitative Analysis on Developing Korean Disaster Mental Health Support Model

**DOI:** 10.3390/ijerph15020362

**Published:** 2018-02-18

**Authors:** Yun-Jung Choi, Hwa-Bok Choi, Meaghan O’Donnell

**Affiliations:** 1 Red Cross College of Nursing, Chung-Ang University, Seoul 06974, Korea; ghkqhr83@naver.com; 2 Phoenix Australia, Centre for Posttraumatic Mental Health, University of Melbourne, Melbourne, VIC 3002, Australia

**Keywords:** disaster, mental health, posttraumatic stress disorder, psychological recovery, qualitative research

## Abstract

This study sought to describe the mental health problems experienced by Korean disaster survivors, using a qualitative research method to provide empirical resources for effective disaster mental health support in Korea. Participants were 16 adults or elderly adults who experienced one or more disasters at least 12 months ago recruited via theoretical sampling. Participants underwent in-depth individual interviews on their disaster experiences, which were recorded and transcribed for qualitative analysis, which followed Strauss and Corbin’s (1998) Grounded theory. After open coding, participants’ experiences were categorized into 130 codes, 43 sub-categories and 17 categories. The categories were further analyzed in a paradigm model, conditional model and the Disaster Reintegration Model, which proposed potentially effective mental health recovery strategies for disaster survivors, health providers and administrators. To provide effective assistance for mental health recovery of disaster survivors, both personal and public resilience should be promoted while considering both cultural and spiritual elements.

## 1. Introduction

A growing body of research speaks to the psychosocial impact of trauma and its consequences for mental health services [1]. Like other countries, Korea has experienced catastrophic disasters, which have grown continually in recent years. For example, 502 people died in a shopping center breakdown in 1995, 192 people died in a subway fire in 2003 and 304 died when a ferry submerged in 2014 [2,3]. After the 2003 subway fire, mass media actively reported on survivors’ mental health problems, which included Hwa-Byung (Korean anger disorder), depression, suicide attempts and psychotic behaviors [4]. From those tragic experiences, nationwide demand for a systematic approach to mental health recovery from traumatic incidents increased. From 2003 to 2006, a government-based disaster mental health support system began development nationwide. 

Although social awareness and demand for disaster mental health support have become common as a result of these efforts, Korea’s human and physical infrastructure for disaster relief support services remain in short supply [2]. Disaster mental health support interventions, including models for disaster mental health recovery, have not yet been demonstrated as consistently effective for Koreans [5]. The problems, raised by disaster mental health support researchers after the Seowall ferry disaster in 2014, include not only systematic difficulties related to administration and enforcement but also a lack of standardized education and manuals, doubt about the appropriateness and effectiveness of disaster psychological support services and lack of practitioner education and capacity management [2,6]. 

Recently there has been recognition that there is a gap in the literature concerning psychosocial support for disaster survivors who develop distress but not psychiatric disorder. There are a small number of programs that have been developed to address this gap. Skills for Psychological Recovery (SPR) was developed in the U.S. and is consistent with empirically supported principles following disaster [7]. The six main skills of SPR are (1) gathering information and prioritizing assistance, (2) building problem-solving skills, (3) promoting positive activities, (4) managing reactions, (5) promoting helpful thinking and (6) rebuilding healthy social connections [7]. The International Program for Adjustment and Resilience (InterPAR) was developed by an international consortium of disaster and mental health experts [8]. and has six components delivered over five sessions (1) managing strong emotions (2) promoting healthy living, (3) coming to terms with the disaster, (4) maintaining healthy relationships, (5) getting back into life and activities and (6) rumination and worry control [8]. Importantly, InterPAR was developed to be delivered by trained volunteers or non-mental health professionals to increase the reach of the intervention after disaster. It is important to assess whether these programs would be relevant to the Korean culture, before they are incorporated into a disaster response plan in Korea [9]. However, there is very little literature that has investigated the subjective disaster psychosocial response by Koreans. 

Qualitative research methods are often used to explore the subject’s experience, to derive variables related to the nature of the experience and to identify relationships between the concepts to develop a substantive theory of the subject’s experience. One qualitative approach, the grounded theory method, is a way to present the systematic steps and processes involved in carrying out this purpose. Grounded theory is appropriate for identifying and analyzing complex and hidden processes [10]. The results of grounded theory are abundant, have a clear form, provide explanations that can express reality and result in creating new knowledge and guiding practice [11]. Thus, in exploring disaster survivors’ experiences and deriving elements of cultural and situational contexts, grounded theory may be an effective method.

The purpose of this study was to investigate in-depth the disaster-related mental health problems experienced by survivors of disasters using grounded theory, a qualitative research method, in order to provide empirical resources on effective disaster mental health support in Korea.

## 2. Materials and Methods 

**Figure 1:**
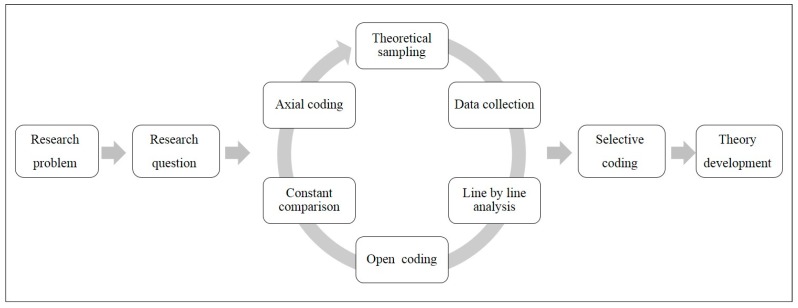
Grounded Theory Flow Chart.

A qualitative research design using the Grounded theory was applied to explore the disaster-related mental health problems experienced by Korean disaster survivors in-depth. The process of grounded theory is illustrated in Figure 1.

Participants were adults (aged 20–64) and elderly adults (aged over 65) who had experienced one or more disasters at least 12 months ago. The definition of disaster was taken from the Korean Disaster and Safety Law that includes natural, social and manmade disaster. The participants were recruited by posting advertisements on bulletin boards of the Korean Red Cross Society, firefighting schools, public health centers and welfare centers and the number and scope of participants was expanded using the snowball method. The inclusion criteria were adults aged over 18 who were able to verbal communication and the exclusion criteria were adults who had acute health problems. In the Grounded theory research method, the number of participants is not specified in the research plan stage but the same data are repeated throughout in-depth interviews. When a certain pattern is repeated and no new expressions emerge from the participants, it is considered “data saturation,” at which point data collection is concluded [10].

Data collection for the Grounded theory research is conducted by two research assistants who had experiences of practice or education regarding disaster mental health services and qualitative studies. They collected data through interviews with research participants, field notes and memos. To understand effective mental health management strategies experienced by disaster survivors in the disaster response process, we generated the research question, “What is the process of effective mental health management of the disaster-experienced person?” Interviews with participants were recorded and memos were written to capture non-verbal expressions such as laughter, tears, sighs and posture. 

The interviews were conducted in counseling rooms at community mental health centers, participants’ homes, or other locations that ensured the participant’s privacy. Each participant was interviewed once or twice and each interview lasted 60–90 min. Each entire interview was recorded digitally and transcribed verbatim immediately after the interview. Memos and field notes on participants’ non-verbal communication as well as the researcher’s thoughts or impressions were recorded as additional data for analysis.

The analysis procedure of the data followed the steps of grounded theory analysis [10]. The first step in the theory-based analysis is coding. We analyzed and conceptualized data and recombined them in a new way. Open coding is an analytical process that identifies concepts and evolves according to attributes and dimensions. We categorized similar results or incidents by comparing similarities and differences between instances according to phenomena. Axial coding was the second step of analysis. We verified the relationships between subcategories against the data, including a set of procedures, conditions, contexts, strategies and results that reconciled the data in a new way after open coding, creating associations between categories. Finally, a model was developed that included the causal conditions, the context, the interventional situation, the action/interaction strategy and the outcome derived by using the form of coding. In the process of conducting the study, the researchers clarified what their interests were and what their preunderstandings were that affected results of the study.

### Ethical Approval

This study was approved by the institutional review board of the researchers’ organization. The ethical approval code (IRB number) of our study is 1041078-201704-HR-070-01. The participants were explained the research purposes and process and were informed that they could refuse or withdraw participation at any point, without detriment. Participants who understood the study conditions and signed the study consent forms were allowed to participate in the study.

## 3. Results

A total of 16 disaster survivors (5 males and 11 females) participated in the study Most participants were married and half were educated at the high school or university level. Participants experienced disaster between 1 and 10 times. Among the disaster types, 12 were floods, 3 were traffic accidents and 1 was a fire (Table 1).

### 3.1. Results from Open and Axial Coding

**Figure 2:**
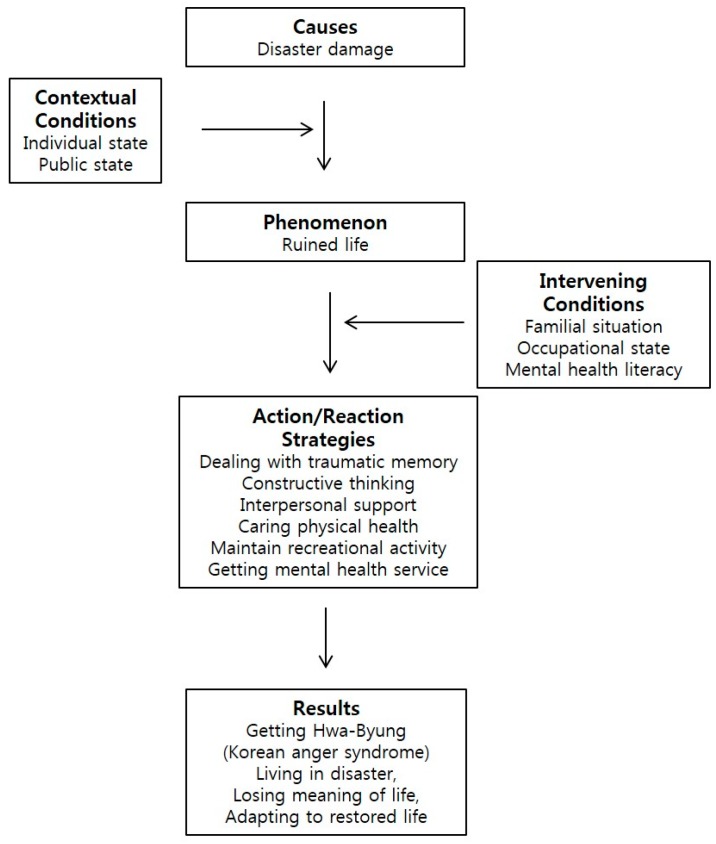
Korean’s coping process for mental health recovery from disaster.

After open coding analyses, mental health problems were categorized into 130 codes, 43 sub-categories and 17 categories. The categories were further analyzed using axial coding, which illustrated in Figure 2.

#### 3.1.1. Causal Conditions of the Disaster Survivors’ Mental Health Problems 

In Grounded theory method, “causal conditions” are events that cause research phenomena to occur. Causal conditions for the disaster survivors’ mental health problems were identified as “disaster damage.” The category included the subcategories “flooding,” “fire,” and “traffic accident.”

All the disaster survivors in our study reported having had an unexpected flooding, fire, or traffic accident, which happened suddenly such that they could not cope using typical responses.

“The liquidation dam had collapsed. Yeah and there’s a backflow here. At that time, two people died in B-dong and three people including Kim were dead. One was swept in the water and one died in an electric shock…”

*(Case 8)*

“I was hit by a fire but the fire accident caused a liquefied petroleum gas explosion. Oh, that’s when the explosion happened and suddenly something happened at that time. I was so embarrassed that I did not know what to do…”

*(Case 2)*

“The feeling that the car itself rolls like this. Because I bumped too much from the back, I was just stuck against it at a speed of 80–90 km/h. So, I was frozen still and in shock, so shaken now, all the junk in the car bounces out as it shakes…”

*(Case 14)*

#### 3.1.2. Contextual Conditions of the Disaster Survivors’ Mental Health Problems

“Contextual conditions” are any situation that affects research phenomena. In this study, contextual conditions of the disaster survivors’ mental health problem experiences were identified as “individual state” and “public state.” Three sub–categories fell under the Individual State Category which included “economic loss,” “physical impairment” and “repetitive flooding.” Disaster survivors suffered from considerable emotional distress because of significant economic loss or physical impairment.

The public support category included the subcategories “dissatisfied with public support” and “receiving public support.” Generally, there was a sense of anger about not receiving economic compensation and a lack of adequate administrative support. Survivors did, however, recognize that they were helped by medical support, housekeeping helpers, or volunteer help.

“If it rained, it would be immersed in water. No matter how much it recovered, it was useless and helpless.”

*(Case 5)*

“I was hospitalized at the hospital with burns on my arms and legs, the pain was so severe that I could not tell.”

*(Case 2)*

“I went on a motorcycle and turned left and I ran into a car making a U-turn. I just rolled up and fell back like this. My neck was broken and my hands and feet were swollen by the shock but my feet were walking fast but my hands were so swollen that I could not touch them.”

*(Case 15)*

“If you give me a chance by economic support to recover quickly, or if you give me a chance to recover soon, I will be better off.”

*(Case 13)*

#### 3.1.3. Phenomenon of the Disaster Survivors’ Mental Health Problems 

The “phenomenon” is exactly what is happening in the subjects' experience. The main phenomenon of this study involved in the disaster survivors’ experiences was “ruined life,” which included the subcategories “psychological pain,” “physical trauma,” and “impairment of daily living.” 

Disaster survivors experienced physical pain such as sleep disorders, musculoskeletal diseases and chronic fatigue and psychological distress such as frustration, sadness, depression, sadness and suicidal ideation.

“After recovery, I came back home and I was in a state of being involved with the dirt all over the house. It was so terrible. Oh, now, one by one, now my hard work was broken that I had built throughout my life and it feels like it’s collapsing; how do I do this? I feel like I have lost a lot.”

*(Case 4)*

“No, I want to die even if I live, I just do not want to live...I just want to die. I have no idea. Yes. I do not have anyone in my ears. I cannot express the real condition that I faced…”

*(Case 1)*

“At that time, I could not think of anything at the time. Just because I was flooded, I just cried and was blowing, so I fell asleep and cried like hell. At that time, I could not think to ask for help.”

*(Case 11)*

#### 3.1.4. Intervening Conditions of the Disaster Survivors’ Mental Health Problems 

“Intervening conditions” are any situation that affects the strategy to cope with research phenomena. In this study, intervening conditions of the disaster survivors’ mental health problem were analyzed under the categories “familial situation,” “occupational state,” and “mental health literacy,” which included the subcategories “receiving support from family,” “considering familial condition,” “maintaining occupation,” “lost one’s job,” “urgency of recovery,” and “understanding mental health.”

Disaster survivors who received assistance from their families recognized this as part of a restoration process. Other participants were concerned with burdening their children as a result of the disaster and sought to protect their family from negative effects of the disaster. Even in a disaster situation, maintaining a job helped in recovery but in some cases, they had difficulty maintaining their jobs because they had not received adequate support or treatment. Some people commented that they did not receive mental healthcare services because the government response concentrated on the physical aspects of disaster recovery and the focus on mental healthcare was limited.

“I think I should save my child. Because I am a mother. The damage was huge. And should we do this again? But it was because things started out so hard but I am a mother and I have to live…”

*(Case 10)*

“There is a limit to outsiders’ ability to help and they are busy too. So, first of all, family members gave a lot of help, primarily when things were so bad.”

*(Case 9)*

“I stopped receiving medical treatment because I felt the gaze of my colleagues when I visited the hospital, even though I should be treated after the flooding.”

*(Case 6)*

“I do not know where to go for psychological treatment or something like this but at home and outpatient treatment, so to speak, I would say it is a folk remedy, so I tried to do something like that.”

*(Case 2)*

#### 3.1.5. Action/reaction Strategies of the Disaster Survivors’ Mental Health Problems 

“Action/reaction strategies” of the Grounded theory are the means used to manipulate and control the “phenomenon.” Action/reaction strategies for the disaster survivors’ mental health problem fell under the categories “dealing with traumatic memory,” “constructive thinking,” “interpersonal support,” “caring for physical health,” “receiving mental health service,” and “maintaining recreational activity.” Subcategories of these strategies included “journaling,” “concentrating on simple activities,” “volunteering,” and 15 other sub-categories (Table 2).

The disaster survivors struggled to live with a positive mind and managing their own minds in coping with the “ruined life,” the phenomenon of in this study, with support from family and colleagues and with their communities. In addition, they tried to find psychological stability through psychotherapy, by taking psychiatric medication or psychological counseling. Some of them sought traditional therapy for their own physical health and mental problems. They also used strategies to cope with religious life, hobbies and regular exercise.

“Now, I start driving and praying…Sometimes when the car changes next to the lane, it also jumps slightly. Every time I say to myself...I’m fine, I am much better and now I’m okay. I am safe. I always keep talking to myself like this.”

*(Case 14)*

“I exercise a lot. Before exercising in the morning, I run for 1 hour. Exercise reduces sick days and decreases the number of times I go to the hospital. That’s why I like exercise.”

*(Case 3)*

“Because I went on without quitting my job, I forgot about the accident. I think that the mental suffering was a little less because my colleagues and I have been working this way now…”

*(Case 2)*

“Actually, we are helping each other and living comfortably with the neighbors. If you live with each other in a spirit of cooperation, it seems that you can overcome many problems.”

*(Case 7)*

#### 3.1.6. Results of the Disaster Survivors’ Mental Health Problems 

In Grounded theory, “results” are the consequences of interaction with the phenomenon. The results of the disaster survivors’ mental health problem experiences were categorized as “living in disaster”, “losing meaning of life”, “getting Hwa-Byung (Korean anger syndrome)”, and “adapting to a restored life”. Subcategories of the results included “chronic health problems”, “chronic physical symptoms”, “being powerless”, “despair”, “no meaning of life”, “accepting reconstructed environment”, “adjusting self to changed situation”, “re-experiencing disaster”, and “avoiding disaster situation”.

Most of the disaster survivors were found to suffer from Hwa-Byung (Korean anger syndrome) after the disaster, as well as chronic symptoms such as cerebral infarction. In addition, there were cases in which the meaning of life was lost, such as being helpless or experiencing self-abandonment with repetitive flooding. On the other hand, some of the survivors lived and rebuilt their lives after discovering the meaning of life. Some of them could not sleep or were afraid of another flood when it rained and they avoided burning gas or tried to avoid trauma.

“When it rains, I become anxious now. I cannot go far away. I wonder what would happen if I went there. If something goes away when you’re far away, that’s it”.

*(Case 10)*

“Every time I see a scar, the pain of the time comes up, I hate to meet people. I just think I do not want to live like this”.

*(Case 2)*

“I’ve got Hwa-Byung. If I get nervous, the symptoms are already building inside. I cannot just breathe. If I just get sick in the abdomen, I must run to the toilet. After the flooding, my body is bad and hardi”.

*(Case 9)*

“The most helpful strategy was to live hard. Because I should do my job. We do not want to rely on another person. It’s just living hard that is medicine”.

*(Case 3)*

### 3.2. Results from Selective Coding

#### 3.2.1. Core Category: Rebuild and Rehabilitation

**Figure 3:**
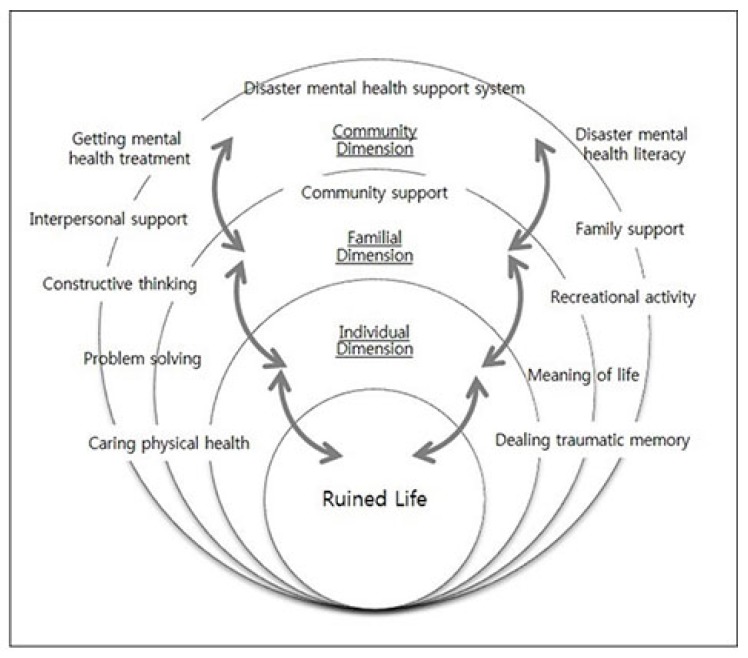
The conditional matrix of Korean’s coping process for mental health recovery from disaster.

In this study, the core category, comprising all other categories of the disaster survivor’s experience of mental health problems, was defined as “rebuild and rehabilitation”. Disaster survivors in this study used family coping strategies to cope with the phenomenon of “ruined life” in the process of rebuilding and rehabilitating. At the community level, participants went to a religious institution and devote themselves to their faith or overcome difficult situations by living together with their community and peers. Figure 3 represents the coping process of “Ruined Life”, tracking how the outcome varies according to the various conditions of interaction and interaction with each conditional situation at various levels such as individual, familial and community dimensions.

#### 3.2.2. Conceptual Framework: Disaster Reintegration Model

**Figure 4:**
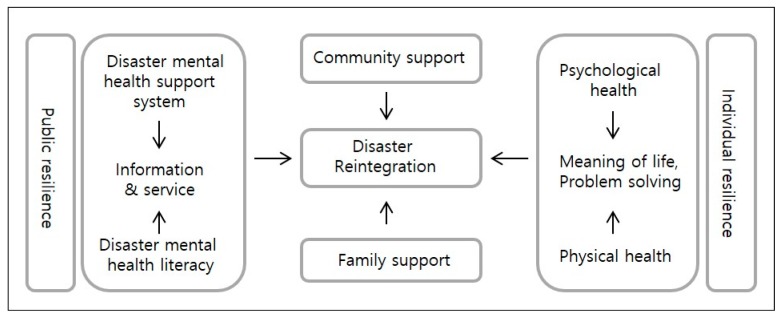
Disaster reintegration model.

The conceptual framework described above was integrated into a “Disaster Reintegration Model”, which incorporates all categories, subcategories and codes and explains the storyline explored in this study (Figure 4). The model included 12 final concepts: “Disaster reintegration”, “Public resilience”, “Individual resilience”, “Physical health”, “Psychological health”, “Meaning of life”, “Problem solving”, “Family support”, “Community support”, “Disaster mental health support system”, “Information and service” and “Disaster mental health literacy.” This model explains that supporting the disaster resilience of individuals and the public are important in rebuilding and resurrecting from disaster impacts. In order to support “individual-level resilience”, “psychological health”, “physical health” and “problem-solving ability” is promoted and “meaning of life” is discovered. To support the “public dimension of resilience”, it is necessary to establish a “disaster mental health support system”, promote “disaster mental health literacy” and provide effective “disaster mental health services and information”.

## 4. Discussion

This study was conducted to explore mental health recovery process and strategies for disaster survivors using qualitative research methods. From the results of present research, we derived factors from hypothetical models related to disaster mental health recovery to describe effective strategies for mental health recovery after the disaster. In this section, we review cultural aspects of Korean disaster survivors on the paradigm model and discuss mental health recovery strategies and conceptual models to suggest basic resources for developing a Korean disaster mental health support program.

Both SPR and InterPAR include techniques for managing emotional reactions related to the disaster experience such as “breathing exercise” and “grounding techniques,” which are evidence-based strategies for disaster mental health support. Participants of this study did not indicate using these strategies to cope with their negative emotional responses, though some received professional health services for their physical or mental health problems after the disaster. This is related with the low level of disaster mental health literacy among health professionals in Korea, which needs to be increased through education on disaster mental health support programs and then certified for professional service. 

In this study, the disaster survivors experienced various types of psychological pain, physical trauma and inability to perform daily living in the acute stage after the disaster. Over time, as their symptoms progressed into the chronic stage, most participants reported that the symptoms turned into Hwa-Byung, a kind of Korean anger syndrome included in the DSM-IV as a culture-bound syndrome [4,12], which was one of the categories of the paradigm model in this study. A person with Hwa-Byung is often diagnosed as depression, anxiety disorder, or somatic disorder [12]. The prevalence rate of Hwa-Byung is estimated from 4.2% to 11.9% in Korea [4]. Symptoms of Hwa-Byung manifest as various physical-psychological symptoms, where somatization arises through unresolved anger [12]. Therefore, to manage traumatic stress effectively for Koreans, a disaster mental health support program must include both anger management and caring for physical health. Though some researchers have reported that cultural factors would not be important in providing mental health support after disaster [13], results of this study propose the need of delivering disaster mental health recovery support in a culturally sensitive way that is in line with a previous study [14].

Thus, the results of the present study included the category “caring for physical health” among the eight action/reaction strategies of the disaster survivors’ mental health problems. This is consistent with the element of “promoting healthy living” of InterPAR, which also suggests a holistic approach to disaster mental health support [8]. The participants of this study used strategies to cope with traumatic stress such as positive thinking, mind control, empowering the self, regular exercise and using hobbies to relieve stress, which was categorized as “constructive thinking” and “recreational activity.” Those coping strategies are in line with “promoting helpful thinking” and “promoting positive activities,” which are among the main skills of SPR [7]. It is also consistent with a number of the aspects of InterPAR which include promoting regular exercise and emotional regulation activities such as present-centered awareness and relaxation training.

The disaster survivors of this study used several strategies for “dealing with traumatic memories,” also in line with the elements of SPR and InterPAR. For example, they wrote in journals to organize thoughts or concentrated on simple activities such as quilting, which are included in InterPAR among its key components, “coming to terms with the disaster” and “rumination and worry control” [8]. Volunteering is suggested in “rebuilding healthy social connections” as skills of SPR [7], which was also reported by participants of the present study as an effective coping strategy. InterPAR correspondingly recommends maintaining healthy relationships for disaster survivors, in accordance with the category “interpersonal support” derived from this study [8]. Some of the participants expressed “maintaining occupation” as an effective intervention to distract from potential rumination over their disaster experiences and receiving support from colleagues. This is similar to a key component of InterPAR, “getting back into life and activities,” which protects against adjustment problems in daily life. 

As the above discussion indicates, it is important to increase individual resilience of the disaster survivors to promote mental health recovery. The Disaster Reintegration Model derived in the present study emphasizes not only personal resilience but also public resilience, proposing to provide adequate information and services for disaster mental health support as well as to improve disaster mental health literacy [15]. In this study, “meaning of life” was included in the conceptual model with “rely on religion,” which are among the action/reaction strategies that are in line with “activities that have importance to you” of InterPAR [8]. This suggests that spiritual approach should be considered in helping disaster survivors. It is suggested to apply the frameworks derived from this study to a larger population in various cultural backgrounds. Further studies to measure relationships among the concepts of the Disaster Reintegration Model would be recommended.

## 5. Conclusions

The present study proposed a paradigm model, a conditional matrix and the Disaster Reintegration Model derived from experiences of disaster survivors regarding effective strategies for their mental health recovery. In order to promote mental health recovery of disaster survivors, it is necessary to promote both personal and public resilience while considering cultural and spiritual factors.

## Figures and Tables

**Table 1:** General characteristics of the participants.

No.	Sex	Age (Year)	Status of Marriage	Education	No. of Disaster Experience
1	Female	73	Divorced	Middle school	2
2	Female	67	Married	High school	1
3	Female	77	Married	Middle school	3
4	Female	45	Married	University	1
5	Female	76	Bereaved	Middle school	1
6	Female	81	Bereaved	High school	4
7	Male	75	Married	Middle school	3
8	Female	44	Married	University	2
9	Male	66	Married	High school	10
10	Male	57	Married	High school	2
11	Female	64	Married	College	5
12	Female	59	Bereaved	Middle school	1
13	Female	57	Married	Middle school	10
14	Male	67	Married	High school	10
15	Male	51	Married	High school	3
16	Female	37	Single	College	2

**Table 2:** Results of open coding and axial coding from the disaster survivors’ experiences.

Open Coding		Axial Coding
Sub-Category	Category	
Flooding	Disaster damage	Causal Conditions
Fire		
Traffic accident		
Economic loss	Individual state	Contextual Conditions
Physical impairment		
Repetitive flooding		
Dissatisfied with public support	Public state	
Receiving public support		
Physical Trauma	Ruined life	Phenomenon
Psychological pain		
Impairment of daily living		
Receiving supports from family	Familial situation	Intervening Conditions
Considering familial condition		
Maintaining occupation	Occupational state	
Lost one’s job		
Urgency of recovery	Mental health literacy	
Understanding mental health		
Journaling	Dealing traumatic memory	Action/Reaction Strategies
Concentrating on simple activities		
Rely on religion		
Positive thinking	Constructive thinking	
Mind control		
Self-empowerment		
Recognizing interpersonal relationship	Interpersonal support	
Receiving community support		
Volunteering		
Combining traditional remedies	Caring physical health	
Managing physical health		
Having physical treatment		
Prescribed psychotropic medication	Getting mental health service	
Receiving psychological counseling		
Seeking professional information		
Ventilating by hobby	Recreational activity	
Exercise regularly		
Chronic health problems	Getting Hwa-Byung (Korean anger syndrome)	Results
Chronic physical symptoms		
Being powerlessness	Losing meaning of life	
Despaired life		
No meaning of life		
Accepting reconstructed environment	Adapting restored life	
Adjusting self to changed situation		
Re-experiencing disaster	Living in disaster	
Avoiding disaster situation		
